# Role of transpalatal advancement pharyngoplasty in management of lateral pharyngeal wall collapse in OSA

**DOI:** 10.1016/j.bjorl.2021.04.009

**Published:** 2021-05-06

**Authors:** Ahmed Elsobki, Waleed Moneir, Mohamed Abdelbadie Salem, Mohamed Elkahwagi

**Affiliations:** ORL, HNS and Maxillofacial Surgery, Mansoura University, Mansoura, Egypt

**Keywords:** Transpalatal advancement, Lateral pharyngeal wall, Collapse, Hypopharynx, OSA

## Abstract

**Introduction:**

Non-response to palatal surgery for OSA is a problem. Residual lateral wall hypopharyngeal collapse is the proposed mechanism of failure.

**Objective:**

This study aims to evaluate the role of transpalatal advancement pharyngoplasty in non-responders to primary palatal surgery with residual lateral wall hypopharyngeal collapse.

**Methods:**

This is a retrospective study that was conducted on patients who underwent transpalatal advancement pharyngoplasty for residual lateral wall hypopharyngeal. Inclusion criteria were age greater than 18-years, OSA proved by the polysomnography with apnea hypopnea index >15, lateral wall collapse at the level of hypopharynx as proved by drug-induced sleep endoscopy and had a previous tonsillectomy or previous palatal surgery for OSA. Exclusion criteria were those with no history of tonsillectomy or any other surgery for OSA and those with a missed followup. Data of included patients were collected and included gender, age, polysomnographic data like the apnea hypopnea index, oxygen desaturation and the calculated preoperative Epworth sleepiness scale. The early outcome included symptom improvement as measured by Epworth sleepiness scale score and lateral pharyngeal wall evaluation by nasopharyngoscopic examination in the first postoperative month. Late outcome measurement was performed by the 6-month postoperative polysomnography. Data were analyzed using SPSS program.

**Results:**

The study included 37 patients with a mean age of (40.43 ± 6.51). The study included 26 men and 11 women. There was a statistically significant improvement of apnea hypopnea index from 37.8 ± 9.93 to 9.9 ± 2.55. In addition, a statistically significant improvement of lowest oxygen saturation from 78.9 ± 3.39 to 83.3 ± 3.31 was encountered. The patients improved clinically, and this improvement was measured by statistically significant improvement of Epworth sleepiness scale score and snoring visual analogue scale.

**Conclusion:**

Transpalatal advancement pharyngoplasty widens the retropalatal airway and has a great role in the management of the vertical palate phenotype. In addition, it can have a role in the management of lateral walls, especially lateral wall hypopharyngeal collapse.

## Highlights

• Transpalatal advancement pharyngoplasty is the procedure of choice for management of vertical palate phenotype.
• The study highlights a new role for transpalatal advancement pharyngoplasty in strengthening of lateral pharyngeal walls.
• Non- responders to primary palatal surgery for obstructive sleep apnea showed 75% success after transpalatal advancement pharyngoplasty.
• Multilevel collapse at the palate and hypopharyngeal lateral wall is a good indication for transpalatal advancement pharyngoplasty.

## Introduction

Obstructive sleep apnea (OSA) is a multifactorial disease with many pathophysiologic mechanisms that are responsible for the airway collapse during sleep.^1^ The main causes likely include an abnormality in the upper airway anatomy and the responsiveness of the upper airway dilator muscles to respiratory events during sleep.^2^ Targeting the treatment to the patient’s pathology broadens the treatment options and improves patient acceptance and surgical results.^3^

The accurate diagnosis of the site of the airway collapse in OSA is the key to the success of the management plan.^4^ Tailoring the treatment plan to each patient is ideal rather than applying a standardized approach for all patients of OSA.^5^ Endoscopic upper airway examination has become the cornerstone in the evaluation of OSA patients.^6^ Drug-induced sleep endoscopy (DISE) is now the most practical tool for diagnosis of the collapse site and can be considered the driver tool of the management plan.^7^

Elsobki et al. proposed a detailed classification system for data gained from DISE that illustrated the segmental division of the lateral pharyngeal wall and described the retropalatal collapse intensively.^1^ They proposed a management plan based on this classification for DISE data.^1^ However, palatal surgery still is the commonest to be currently performed for OSA surgical management.^8^ This may be attributed to the highest incidence of palatal collapse and retropalatal obstruction in OSA patients.^7^, ^9^ On the other hand, palatal surgery in general and uvulopalatopharyngoplasty (UPPP) especially showed a variable success rate from 25% to 80%.^10^ According to recent studies, the success rate of UPPP has declined to 40% especially in moderate and severe OSA.^11^ Enhancing the success rate of palatal and velopharyngeal surgery remains a challenging issue.^12^, ^13^

In a large study by Kerizian which was held on a group of non-responders for primary surgery for OSA, he proposed that residual obstruction at the level of the lateral hypopharyngeal wall (LH) may be the cause for imperfect outcomes after palate surgery.^14^ Lateral pharyngeal wall procedures like lateral pharyngoplasty and expansion sphincter pharyngoplasty may treat the oropharyngeal lateral walls but may not have the optimum effect at the level of the hypopharynx.^14^, ^15^, ^16^ Elsobki et al. proposed that expansion sphincter pharyngoplasty can have a role in the management of LH collapse.^1^ Other surgeons proposed bony framework procedures like hyoid suspension and maxillomandibular advancement for management of LH collapse.^17^, ^18^, ^19^ However, Kezirian found that many of these procedures may have residual collapse at the hypopharynx after surgery.^14^ He argued that hyoid suspension may treat the epiglottis more specifically than the lateral hypopharyngeal walls due to anterior displacement and stabilization of the hyoid bone and, indirectly, the hyoepiglottic ligament.^14^ In addition, Bowden et al. who conducted a prospective study for evaluation of hyoid suspension with UPPP in OSA patients achieved a success rate of only 17% and concluded that hyoid suspension alone is not an effective treatment for a hypopharyngeal collapse in OSA patients.^17^

Woodson and Toohill firstly proposed transpalatal advancement pharyngoplasty (TPAP) in 1993 as a modification of the commonly performed procedure at that time, which was UPPP.^20^ They proposed that TPAP would increase the size of the oropharynx by palatal advancement rather than soft tissue excision.^20^ The procedure was originally described by first performing a “gothic arch” incision to expose the hard palate. This procedure was modified to a propeller incision by Shine in 2007 to decrease the likelihood of creating an oronasal fistula and few modifications of the original procedure have otherwise been made.^21^

This study aimed to evaluate the role of the TPAP in splinting the lateral wall especially at the level of the hypopharynx. We hypothesized that performing the TPAP for the non-responders to primary palatal surgery for OSA or those who even had a previous tonsillectomy, especially those who show lateral wall hypopharyngeal collapse on DISE, would achieve a favorable outcome for the surgery. The specific aim of the study was to evaluate the role of the TPAP in improving the AHI and lowest O_2_ saturation in non-responders to primary palatal surgery with LH collapse.

## Patients and methods

### Study design

To address the research purposes, the authors designed this retrospective study for the obstructive sleep apnea (OSA) cases that had transpalatal advancement pharyngoplasty (TPAP) for lateral wall hypopharyngeal (LH) collapse proved by preoperative drug-induced sleep endoscopy (DISE) and Muller’s manuver. Institutional review board approval for retrospective evaluation and data collection was obtained before initiation of the study (reference number R: RP.20.06.72). The study population was composed of all patients of OSA with LH collapse presenting to Mansoura University Hospitals between January 2014 and January 2019. To be included in the study sample, patients had to fulfill the following inclusion criteria: age greater than 18-years, OSA proved by the polysomnography with apnea hypopnea index (AHI) >20, with lateral wall collapse at the level of hypopharynx (LH) either alone or combined with residual retropalatal collapse as proved by the DISE and had a previous tonsillectomy with a scarred lateral pharyngeal wall or previous palatal procedure for OSA. Exclusion criteria were those with no history of tonsillectomy or any other surgery for OSA and those with a missed follow-up or those who did not complete the follow-up period. Data of included patients were collected and included gender, age, polysomnographic data like the AHI, oxygen desaturation and the calculated preoperative Epworth sleepiness scale (ESS).

Study variables: the predictor variable of the study was the performance of TPAP for cases of OSA with mainly LH on DISE who have scarred lateral pharyngeal wall that is making the lateral pharyngeal wall soft tissue procedures more surgically demanding and less successful. The outcome variables were the resolution of OSA symptoms postoperatively, the observation of well-tensioned lateral pharyngeal wall on one-month postoperative awake nasopharyngoscopy and the correction of the AHI and oxygen desaturation on 6-months postoperative polysomnography.

### DISE technique

For our DISE technique, as we stated before in the paper describing LWPTL classification system for DISE data, the patient was in a supine position on the operating table. The patients had basic cardiorespiratory monitoring (pulse oximetry, blood pressure, and electrocardiogram). The target depth of sedation is the transition from consciousness to unconsciousness (loss of response to verbal stimulation), in practical terms, when the patient started to snore and choke. This description for the desired level of sedation is because we do not have the bispectral index monitoring in our department. Atropine was used for all patients once 30-min before anesthesia at a dose of (0.6 mg/kg) as it is an anticholinergic drug that decreases saliva during evaluation. Sleep was induced using propofol in a dose of (1.5 mg/kg) as a bolus and then maintained with simply manual controlled infusion. Propofol is an ultra-short acting hypnotic that enables greater control of the depth of sedation during sleep endoscopy. So, slow stepwise induction was used to avoid oversedation.^1^

### Surgical approach

**Figure 1:**
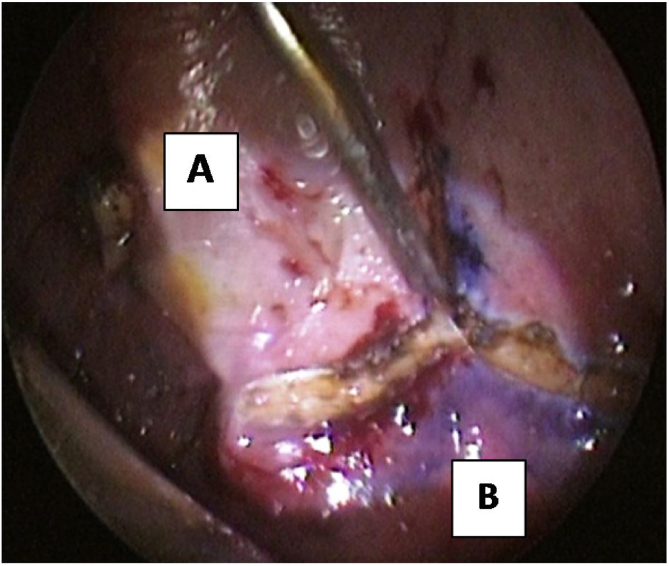
The propeller incision. (A) hard palate; (B) soft palate.

**Figure 2:**
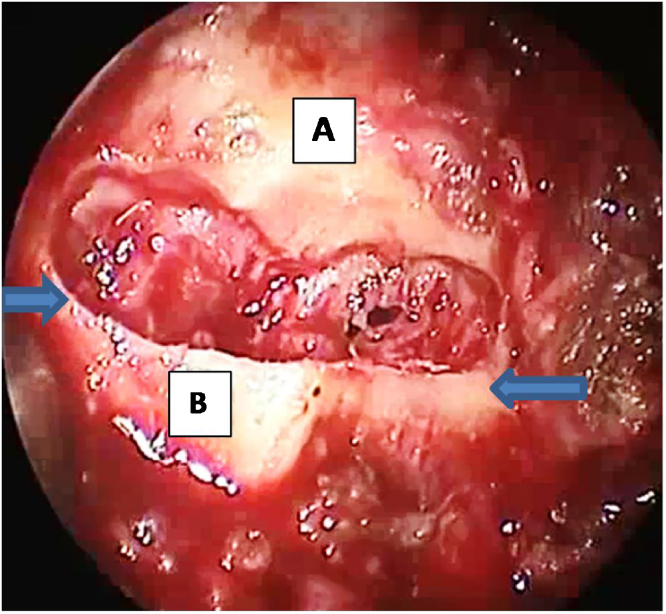
Drilled 1 cm of the posterior hard palate leaving 2-mm of the bone edge and exposing the mucosa without opening it. The arrows refer to the site of the vertical osteotomy to separate the hard and soft palate to enable advancement of the palate, postnasal spine, attached soft palate. (A) hard palate; (B) posterior nasal spine and distal 1-mm of the hard palate.

**Figure 3:**
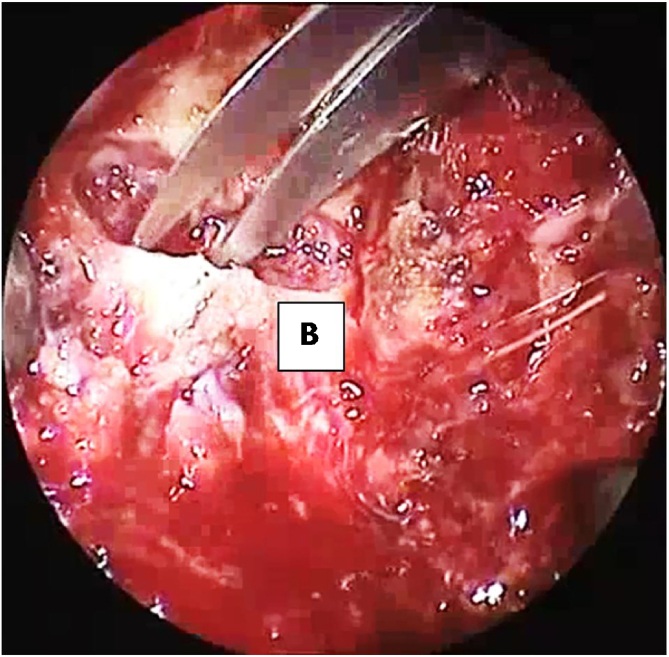
Separation of the soft palate from the posterior nasal septum with heavy scissor. (B) posterior 1-mm of hard palate with attached soft palate.

**Figure 4:**
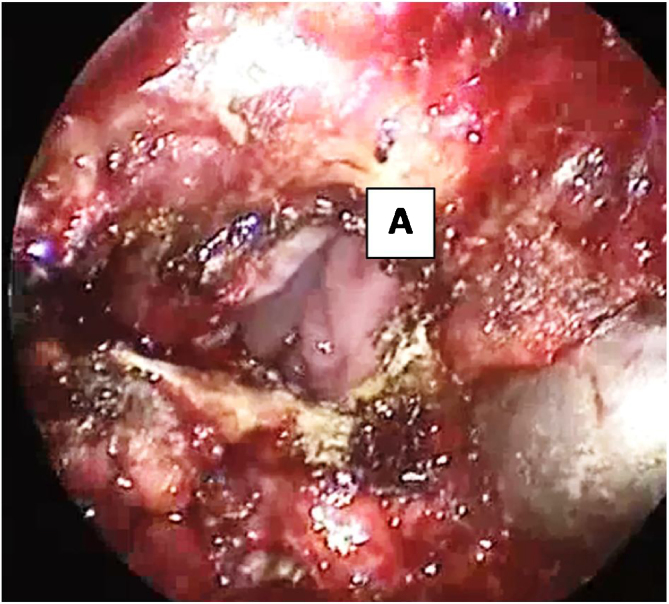
Incising the nasal mucosa and separation of hard and soft palate exposing nasopharynx. (A) nasal mucosa, nasopharynx.

**Figure 5:**
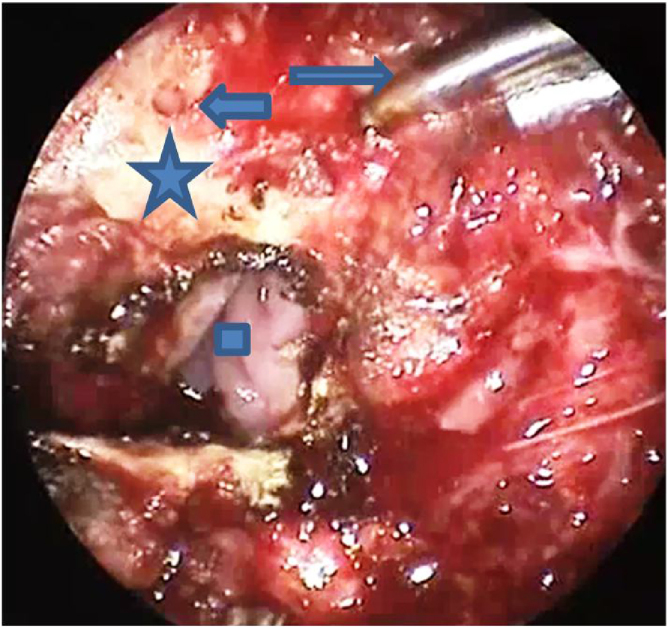
Drill holes in post border of hard palate with the short arrow referring to the hole made on the right side of the posterior hard palate and the long arrow referring to the drill. The square refers to the nasal cavity seen after incision of the mucosa. The star points at the good segment of bone measuring about 4-mm between the drill hole and the edge.

**Figure 6:**
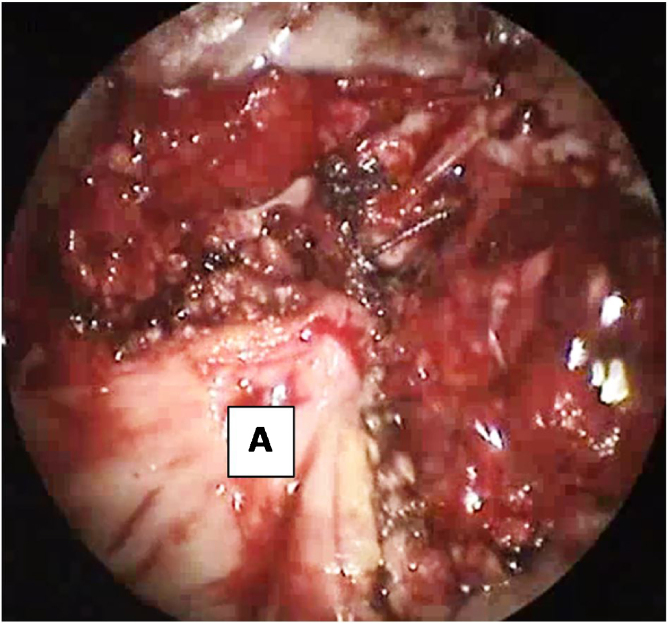
Sutures placed through palatal drill holes into tensor aponeurosis fold laterally. (A) soft palate advanced anteriorly by suturing at the drilled holes at the hard palate.

**Figure 7:**
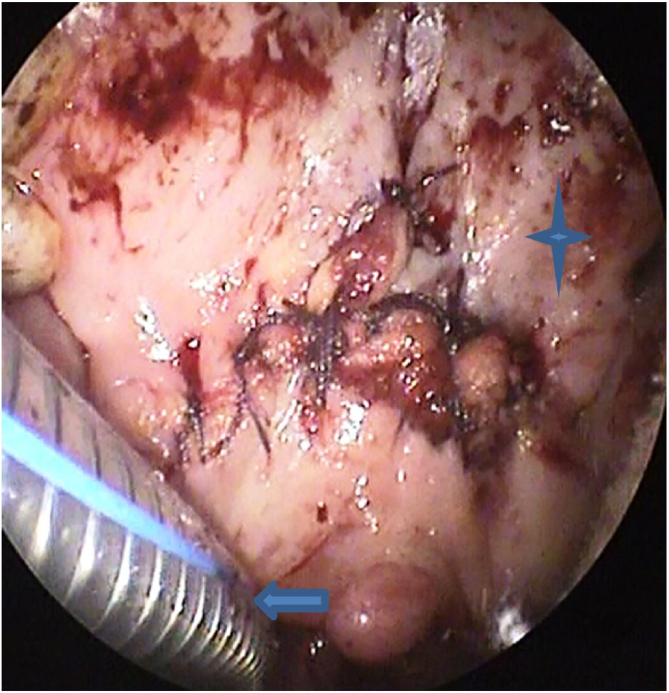
Closure of incision.

The modified approach for TPAP was performed in all cases. The procedure was performed under general anesthesia with oral intubation. For hemostasis, 1% lidocaine with 1:100,000 epinephrine was infiltrated into the exit of the greater palatine foramen, the planned incision sites, the junction of the hard and soft palate, and into the lateral tensor aponeurosis medial to the hamulus. The propeller incision was performed with subperiosteal dissection of the laterally based flaps (Fig. 1). Drilling of the posterior hard palate with a diamond drill until reaching the mucosa was performed, leaving a thin strip of the posterior hard palate (2 mm) with its attachment to the soft palate (Fig. 2). Separation of the soft palate from the posterior nasal septum was accomplished with a heavy scissor (Fig. 3). The bony drilling gives a space to move the soft palate anteriorly. Nasal mucosa was incised (with electrocautery) proximal and lateral to the osteotomy (Fig. 4). Palatal drill holes were placed at a 45° angle to the palate, extending from the oral surface of the palate into the nasal cavity (Fig. 5). A strong segment (3–4 mm) of bone must be left between these drill holes and the excised bony margin. The tensor tendon was incised laterally medial to the insertion on the hamulus. Two vertical osteotomies were placed 2 cm laterally to the posterior nasal spine to completely separate the soft palate attaching to the nasal spine and the posterior rim of the hard palate from the hard palate. Two sutures (braided) were passed through the drill holes into the nasopharynx and around the osteotomy. Sutures were tied while the assistant pulls the palate forward with a curved blunt instrument (Fig. 6). Soft tissue and mucosal closure were performed in two layers to avoid wound dehiscence (Fig. 7).

Outcome measurement: postoperative pain measured by the visual analogue score VAS and improvement of the symptoms of the OSA was the main early outcome in the first five days and the first six postoperative months consecutively and was measured via the improvement of the ESS. Optimum tension of the lateral wall was an early outcome that was measured by the clinical evaluation of the lateral pharyngeal wall by the awake nasopharyngoscopy with performance of Muller’s maneuver one month postoperatively. Improvement of the AHI and oxygen desaturation level was the late outcome measurements as determined by the six months postoperative polysomnography.

### Statistical analysis

Data was analyzed using SPSS (Statistical Package for Social Sciences) version 15. Qualitative data was presented as number and percent. Quantitative normally distributed data was presented as mean ± standard deviation (SD). Ordinal data and Quantitative non-normally distributed data were presented as median and range. Comparison of the data before and after the operation was done using Wilcoxon signed rank test. Comparison of the ordinal data was done using the Mann Whitney test. Comparison of the continuous data was done using Student *t*-test. A 2 tailed p-value <0.05 was considered to be statistically significant.

## Results

Sixty-five patients who underwent TPAP surgery from the period of 2014 till 2019 were identified. All patients had preoperative polysomnography and they all refused continuous positive airway pressure (CPAP) therapy. Twenty-eight patients were excluded from the study. Reasons for exclusion were the presence of tongue base collapse on DISE (8-patients), primary TPAP for tunnel retropalatal airway identified on DISE (10-patients), TPAP for non-responder to primary surgery without LH on DISE (2-patients), patients with no available postoperative polysomnography as patients refused postoperative polysomnography due to symptoms improvement (8 patients). A total of 37 patients were illegible for inclusion in the study population. Twenty-eight cases had combined lateral wall hypopharyngeal collapse and residual retropalatal collapse. The remaining nine cases had isolated LH collapse.

The study included 26 men and 11 women with a mean age of (40.43 ± 6.51). The included patients had a mean AHI of (37.8 ± 9.93), a mean lowest O_2_ desaturation of (78.9 ± 3.39), a median ESS of 16 (13–20), a median snoring VAS 9 with a range of (7–10) and a preoperative BMI of (33.63 ± 3.71)

Postoperative polysomnographic parameters of AHI and lowest SaO_2_, recorded at followup sleep study, were compared with the preoperative values for each patient. Statistical analysis revealed a statistically significant reduction of AHI, snoring VAS ESS improvement and lowest O_2_ saturation improvement (Table 1, Table 2).

**Table 1:** The preoperative and postoperative data of the included patients with their statistically significant differences.

	Preoperative	Postoperative	*p*-Value
AHI	37.8 ± 9.93	9.9 ± 2.55	<0.001 S
O_2_ Nadir (lowest O_2_ desaturation)	78.9 ± 3.39	83.3 ± 3.31	0.01 S
Snoring VAS	9 (7–10)	3 (2–4)	<0.001 S
ESS	16 (13–20)	8 (5–10)	<0.001 S
BMI	33.63 ± 3.71	33.31 ± 3.47	0.09

**Table 2:** Shows sex distribution of the study population and difference in BMI.

Gender	Preoperative BMI	Postoperative BMI	AHI reduction
Males (20)	(35.02 ± 4.12)	(34.26 ± 3.72)	(29.88 ± 9.15)
Females (17)	(32.24 ± 3.31)	(32.01 ± 3.22)	(25.92 ± 7.25)

Postoperative pain was measured using the VAS score assigned by every patient at the first, 3rd and 5th postoperative days. The pain VAS was 7, 6 and 3 consecutively (Table 3).

**Table 3:** The difference between postoperative pain measured by VAS between patients with relocation pharyngoplasty and those with TPAP during 1st, 3rd and 5th postoperative days.

Postoperative day	Relocation pharyngoplasty	TPAP	*p*-Value
1st	9 (8–10)	7 (6–9)	0.001
3rd	8 (6–9)	6 (5–7)	<0.001
5th	5 (4–7)	3 (2–5)	<0.001

As regards the primary procedure for OSA in the study population, there were 20 UPPP, 10 expansion sphincter pharyngoplasty, 5 relocation pharyngoplasty and 2 anterior palatoplasty. All patients had the primary surgery before the TPAP in this study with a mean of 20-months before TPAP. All UPPP procedures were performed in other hospitals. The relocation pharyngoplasties were performed by the authors for retropalatal collapse. The sphincter pharyngoplasties were performed for lateral wall collapse. The anterior palatoplasties were performed for anteroposterior palatal collapse.

According to Sher’s criteria for surgical success in OSA, we had 75% of patients with more than 50% reduction of AHI and postoperative AHI less than 20.

**Figure 8:**
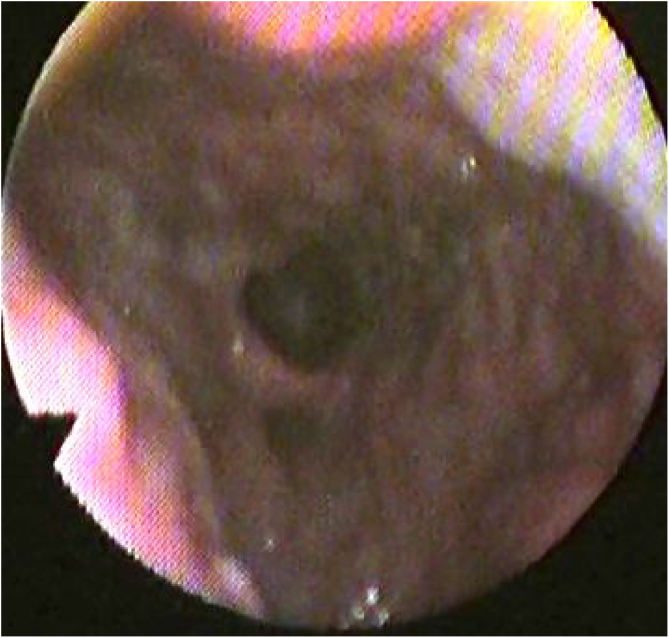
A postoperative nasopharyngoscopy showing wide retropalatal space and well-tensioned lateral pharyngeal walls. The arrow of the camera refers to the soft palate and the froth is on the posterior pharyngeal wall. The arrow refers to the tongue base, the star refers to the posterior pharyngeal wall.

Nasopharyngoscopic examination of the airway one month postoperatively was performed in all patients with a performance of Muller’s maneuver. A wide retropalatal airway was achieved in all patients with good tension in the lateral pharyngeal walls (Fig. 8). We noticed that the more the supported the lateral pharyngeal walls correlate with better improvement of the AHI on 6-months postoperative polysomnography.

No major complications like massive bleeding or death were encountered in the study. The main complication faced in the study was the development of a postoperative oronasal fistula that occurred in 5 patients. The fistula closed spontaneously in 4 patients and required surgical closure in 1 patient. Partial swallowing difficulty was noted in 3 patients and resolved spontaneously within a month postoperatively. No patients developed clinically identifiable changes in speech character or velopharyngeal problems.

## Discussion

Non-response to primary palatal and pharyngeal surgery is one of the limitations of sleep surgery.^14^ Many factors are responsible for failure in sleep surgery.^22^ Missed collapse level, improper splinting of the lateral pharyngeal wall, pathophysiologic abnormality and mouth opening during sleep are the main contributing factors for failure.^14^, ^23^ It has been found surprisingly that most cases of failure of UPPP had a residual collapse at the level of the palate and lateral pharyngeal walls.^22^, ^24^ DISE is the recent most accepted tool for evaluation of the sites of airway collapse in OSA patients and its use changed the treatment protocol for these cases.^25^ In this study, we proposed the use of TPAP for the non-responders to primary palatal surgery when having a Lateral wall Hypopharyngeal (LH) collapse on DISE. Primary surgical failure was defined as patients who have a reduction of the AHI of less than 50% or those who still have the AHI of more than 20.^26^

The concept of the advancement of the palate is based on the pathophysiology of OSA.^24^ Maxillomandibular advancement (MMA) can be considered the most effective multilevel surgery for OSA treatment.^19^, ^27^ It has been documented that the outcomes of MMA regarding sleep quality improvement are comparable to those of CPAP.^28^ Liu et al. have demonstrated that the change of the pattern of airway following MMA is most evident at the level of the lateral pharyngeal walls.^19^ They documented that patients with most improvement of the lateral wall collapse showed the largest reduction of the AHI postoperatively.^19^, ^29^ However, the MMA is not widely performed due to many reasons, the most important of which are the high morbidity of the surgery and the high technical demands of the procedure.^24^ On the other hand, TPAP advances the soft palate with its attachments of the muscles of the lateral pharyngeal walls through posterior maxillectomy and soft palate advancement. In this procedure, retropalatal airway size is enlarged without altering the facial skeleton and it is mainly directed to the collapse site at the level of the palate.^21^, ^30^ In this study, we propose the role of TPAP not only at the level of the palate but also at the level of the lateral pharyngeal walls especially the level of the hypopharynx. The explanation for that theory is that the lateral pharyngeal wall is composed of pharyngeal muscles like the palatopharyngeus which are proximally originating from the hard palate and palatal aponeurosis of the soft palate and inserting at the thyroid cartilage, forming a notable projection at the lateral pharyngeal wall.^16^ Therefore, theoretically, when the soft palate is advanced anteriorly, this should cause more tension on the lateral pharyngeal wall preventing its collapse in OSA patients.

A recent meta-analysis performed on TPAP showed that this procedure significantly improves the AHI and enlarges the airway at the level of the nasopharynx and oropharynx regardless of the preoperative obstruction site.^31^ They showed that TPAP has significantly improved the AHI from a mean of (54.6 ± 23.0) to a mean of (19.2 ± 16.8) postoperatively.^31^ Woodson proposed TPAP which is based on soft tissue advancement as a potential alternative to UPPP which is based on much soft tissue excision.^20^ The procedure showed a dramatic postoperative improvement on its initial application on 11 patients with improvement of the RDI from 73.3 to 25.1 h.^20^ In this study, we performed the modified TPAP on 37 patients who did not have much improvement of their OSA after primary palatal surgery and showed LH collapse as a cause of their residual symptoms identified by preoperative DISE. We encountered a significant improvement of AHI from (37.8 ± 9.93) to (9.9 ± 2.55) postoperatively.

In comparison of our achieved reduction of the AHI postoperatively with that achieved in hyoid surgery for hypopharyngeal collapse and MMA, the reduction achieved seems to be comparable to these procedures. A significant AHI reduction with a mean and SD of (27.9 ± 8.17) was achieved in our study with significant improvement of the AHI from (37.8 ± 9.93) to (9.9 ± 2.55). This result is comparable to that achieved by hyoid myotomy and suspension achieved by Ong et al. who had AHI reduction from (49.9 ± 16.60) to (29.1 ± 24.9) postoperatively.^32^ The main difference is that they had more severe OSA patients as they utilized the hyoid surgery as a primary surgery for OSA with hypopharyngeal collapse.^31^ In our study we achieved surgical success according to Sher’s criteria of more than 90% as stated in the results.^26^ Indeed, MMA achieved the best AHI reduction from (60.0 ± 25.6) to (7.5 ± 3.4) postoperatively. However, our AHI reduction is comparable to that achieved in MMA even if less reduction is achieved taking into consideration the lower morbidity of the TPAP in comparison to MMA.

The lowest O_2_ saturation is another parameter that improves following TPAP.^12^ Of all the parameters measured using polysomnography, oxygen desaturation level and duration remained one of the most important independent predictors of long-term adverse effects on the cardiovascular and neurocognitive functions.^19^ The meta-analysis performed on TPAP showed significant improvement from (81.9 ± 8) to (85.4 ± 6.9) postoperatively. Woodson did not encounter a significant improvement of the lowest O_2_ saturation.^20^ He argued that the lowest O_2_ saturation may not have such clinical importance as that of the time of having O_2_ saturation less than 85%.^20^ However, Liu et al. documented that the best improvement of the lowest oxygen saturation postoperatively is intimately related to the degree of the tension and the lowest collapsibility of the lateral pharyngeal walls.^19^ In our study, we encountered a statistically significant improvement of the lowest O_2_ saturation from (78.9 ± 3.39) to (83.3 ± 3.31) and this copes with the degree of tension that resulted on the lateral walls.

The meta-analysis performed on TPAP showed that the polysomnographic parameters have improved significantly following the procedure especially the AHI and lowest O_2_ saturation.^31^ However, one of the limitations on gathering the data for metanalysis was that all of the studies discussing the effectiveness of the procedure did not report outcomes for sleepiness, such as the Epworth Sleepiness Scale (ESS) and the snoring improvement.^31^ In our study, we evaluated the clinical outcome of the patients and we had significant improvement of the ESS from a median of 16 and a range of (13–20) to 8 (5–10) postoperatively. In addition, patients reported improvement of their snoring level evaluated by the Visual Analogue Score (VAS) from a preoperative level of 9 (7–10) to a postoperative one of 3 (2–4).

Postoperative pain is one of the nightmares for patients with OSA having any pharyngoplasty procedure. Woodson et al. discussed the postoperative pain in TPAP while comparing that with UPPP and found that the degree of pain and odynophagia following TPAP is much less than that following UPPP.^24^ In our study, we measured the postoperative pain using the VAS determined by the patient at the 1st, 3rd and 5th days postoperatively and the VAS median was 7, 6 and 3 respectively. These values were comparable to the postoperative pain score after relocation pharyngoplasty as an example that was published before^6^ (Table 3). We found that the postoperative pain in TPAP is lower than that of relocation pharyngoplasty with a statistical significance. The authors propose that tonsillectomy and soft tissue procedures of the lateral wall are more painful than TPAP that does not have much mucosal work and dissection.

Despite the relative lack of studies of the TPAP, it has been shown as an effective operation that can be performed as a first stage operation in those who have a multilevel obstruction.^14^ The procedure first was proposed as an alternative to the UPPP by Woodson and Toohil.^18^ With the advancement in the diagnostic procedures for evaluating the airway obstruction sites especially with the practice of DISE in evaluation, it was possible to tailor the surgical approach to every single patient’s pathology.^4^ Since then, the TPAP was indicated for those who have retropalatal airway collapse with vertical palate phenotype.^13^ Woodson 2015 described 3 patterns for palatal morphology: the oblique palate in which the narrowing is at the velum, the intermediate palate in which the narrowing is at the velum and genu and the vertical palate at which the narrowing is at the velum, genu and the hard palate. These patterns were the rationale for selecting the appropriate palatal surgery for OSA especially the TPAP for those with the vertical palate.^30^ Elsobki et al. proposed the TPAP in their treatment algorithm as a surgical suitable procedure for those who have lateral wall hypopharyngeal and high palatal collapse.^1^ The authors still propose the additional role of TPAP in augmenting the lateral pharyngeal walls in a fashion that mimics the tension of the lateral walls provided by the MMA. Therefore, we started performing TPAP for cases with failure of primary palatal surgery having lateral wall collapse at the level of the hypopharynx.

This study shows an emerging role for the TPAP in OSA patients who have lateral wall collapse. It has been shown that a great proportion of OSA patients have multilevel collapse with much proportion of the combined palatal and lateral wall collapse.^1^ Liu et al. have reported a strong association between the degree of collapsibility of the lateral pharyngeal walls and the severity of OSA.^19^ In addition, they documented that the best AHI reduction is usually related to the optimum management of the lateral pharyngeal walls in multilevel surgery for OSA.^19^ Staging of the surgery is the most accepted in surgical management for multilevel collapse to decrease the morbidity for the patients.^21^, ^33^ The authors have the opinion that TPAP could be a good single-stage operation for a great proportion of OSA patients who have multilevel collapse at the levels of the palate and lateral pharyngeal walls. This recommendation needs further studies and validation of the role of the operation in the management of lateral wall collapse.

There are some limitations to this study. Having few studies that discuss the indications and outcome of the TPAP is one of the limitations making the comparison of the outcome of the operation with the literature quite difficult. The retrospective nature of the study is another limitation. Having the operation proposed for a new level of collapse and is dedicated for the non-responders to primary palatal surgery, it was difficult to conduct the study in a prospective fashion. Indeed, more prospective research is needed to validate the role of TPAP in non-responders to primary surgery and even in OSA patients with primary multilevel collapse at both the palate and lateral pharyngeal walls. Lack of manometric studies in our institute didn’t enable us to accurately measure the tension and the space gained in the lateral pharyngeal walls. However, we relied on the postoperative static nasopharyngoscopic evaluation of the lateral pharyngeal walls together with the muller’s maneuver and the improvement of the AHI postoperatively. Propofol infusion manner during DISE performance is another limitation as we did not infuse with a controlled target dose.

## Conclusion

Non-responders to primary palatal surgery enjoy a great benefit on having transpalatal advancement pharyngoplasty. TPAP is the procedure of choice in cases of vertical palate phenotype. In addition, it advances the soft palate anteriorly causing more tension on the lateral pharyngeal wall especially at the level of the hypopharynx. This study is the first to clarify the additional role of TPAP in lateral pharyngeal wall splinting in addition to its primary role in retropalatal collapse. However, more prospective research is needed to augment and clarify this role for TPAP.

## Informed consent

Informed consent was obtained from all individual participants included in the study.

## Financial disclosure

The authors declared that this study has received no financial support.

The study performance was in accordance with the ethical standards of Mansoura University IRB and with the 1964 Helsinki declaration and its later amendments or comparable ethical standards.

## Conflicts of interest

The authors declare no conflicts of interest.
